# On the role of inhibition in suppression-induced forgetting

**DOI:** 10.1038/s41598-023-31063-3

**Published:** 2023-03-14

**Authors:** Kevin van Schie, Jonathan M. Fawcett, Michael C. Anderson

**Affiliations:** 1 Department of Medical and Clinical Psychology, Tilburg University, Tilburg, The Netherlands; 2 Psychology, Education and Child Studies, Erasmus School of Social and Behavioural Sciences, Erasmus University Rotterdam, Rotterdam, The Netherlands; 3 MRC Cognition and Brain Sciences Unit, University of Cambridge, Cambridge, UK; 4 Department of Psychology, Memorial University of Newfoundland, St. John’s, Canada

**Keywords:** Psychology, Human behaviour, Cognitive neuroscience

## Abstract

Suppressing retrieval of unwanted memories can cause forgetting, an outcome often attributed to the recruitment of inhibitory control. This suppression-induced forgetting (SIF) generalizes to different cues used to test the suppressed content (cue-independence), a property taken as consistent with inhibition. But does cue-independent forgetting necessarily imply that a memory has been inhibited? Tomlinson et al. (Proc Natl Acad Sci 106:15588–15593, 2009) reported a surprising finding that pressing a button also led to cue-independent forgetting, which was taken as support for an alternative interference account. Here we investigated the role of inhibition in forgetting due to retrieval suppression and pressing buttons. We modified Tomlinson et al.’s procedure to examine an unusual feature they introduced that may have caused memory inhibition effects in their experiment: the omission of explicit task-cues. When tasks were uncued, we replicated the button-press forgetting effect; but when cued, pressing buttons caused no forgetting. Moreover, button-press forgetting partially reflects output-interference effects at test and not a lasting effect of interference. In contrast, SIF occurred regardless of these procedural changes. Collectively, these findings indicate that simply pressing a button does not induce forgetting, on its own, without confounding factors that introduce inhibition into the task and that inhibition likely underlies SIF.

## Introduction

Every day, stimuli in our world trigger memories of personal events, from the mundane to the emotional. When these reminders bring back unsettling memories (e.g., an emotional break-up or the loss of a loved one) people often intentionally hit a “mental stop button” to halt memory retrieval, a process known as retrieval suppression^1^. Evidence indicates that when people regularly suppress the retrieval of an unwanted memory, these memories become more difficult to recall on later tests^2,3^. Here we examine the mechanisms underlying this mnemonic aftereffect of retrieval stopping, known as suppression-induced forgetting. We are particularly concerned with whether it is truly necessary to “press a mental stop button” to achieve suppression-induced forgetting. Might it be possible, upon confronting a reminder, to perform a simple action, like pressing an actual button, and achieve equivalent forgetting^4^? And if both behaviors cause forgetting, what is the role of inhibitory processes in these approaches?

Retrieval suppression is typically studied with the Think/No-Think (TNT) paradigm^1,5^. In this paradigm, participants first study cue-target associates (e.g., STATUE—PAINT, MUSEUM—VAULT). Subsequently, in the TNT phase, participants only see the cue (e.g., STATUE) and are either instructed to think of the target for cues presented in green (i.e., Think items) or to suppress target retrieval for cues presented in red (i.e., No-Think items). A third set of items (i.e., Baseline items) is studied, but is not presented during this phase. Retrieval suppression in this paradigm is often achieved by one of two general strategies. Participants who receive a direct suppression instruction are asked to avoid thoughts of the target and to purge the unwanted memory from awareness when it comes to mind without replacing it with other thoughts^6–8^; participants who receive the thought substitution instruction, by contrast, are asked to intentionally recall another thought or memory in order to avoid thoughts of the target^6,9^. A surprise final test typically reveals that No-Think items are recalled more poorly than are Baseline items, taken to reflect successful suppression (see for a meta-analysis^10^). Forgetting of No-Think items increases with repeated suppression attempts^1,11,12^, and generalizes across many different types of materials, such as face–scene pairs^13^, word–object pairs^14^, object–scene pairs^15^, even when targets are unpleasant^15–17^.

Stopping retrieval impairs the retention of unwanted memories, in part, by recruiting brain regions in the prefrontal cortex involved in inhibitory control to suppress mnemonic function. Retrieval suppression engages right-lateralized prefrontal control areas, such as the dorsolateral and ventrolateral prefrontal cortices^2^, which have been implicated, via connectivity analysis, in playing a causal role in reducing brain activity in areas involved in memory retrieval (e.g., the hippocampus^6,14,18–21^ and emotional arousal (e.g., the amygdala^19,22^). Behavioral, electrophysiological, and hemodynamic findings using the TNT task have converged to suggest the existence of a domain-general inhibitory control process mediated by the right lateral prefrontal cortex that is critical to stopping actions and thoughts, and to inducing later memory failure for suppressed content^13,21,23,24^.

Evidence for the role of inhibitory control in suppression-induced forgetting also can be found at the behavioral level (for reviews^25,26^). For example, behavioral evidence for inhibition is reflected in the pattern of forgetting on the final memory test. Two types of tests are often used; a Same Probe (SP) test that re-uses the originally studied cue to test recall (e.g., STATUE—?) and an Independent Probe (IP) test that uses a separate cue not seen during retrieval suppression. Many independent probe tests use an extra-list semantic associate of the target (e.g., BRUSH—P ____ for PAINT). The IP test is crucial because it circumvents two non-inhibitory processes that could cause forgetting: associative interference and unlearning^1,27,28^. By the interference account, repeatedly avoiding thoughts about a target (e.g., PAINT) when given a cue (e.g., STATUE) is achieved by generating diversionary associations linked to that cue (e.g., LIBERTY) to prevent awareness of the target; these associations later interfere with target recall when the cue appears at test. Unlearning proposes that repeated suppression weakens the link between the cue (STATUE) and the target (PAINT). Neither of these accounts posits that suppression alters the state of the memory target (e.g., PAINT) itself and both claim that forgetting ought to be specific to testing with the studied cue, a prediction known as cue-dependence. If the target itself is inhibited, however, suppression should render it less accessible not only from the studied cue (STATUE), but also a novel IP Test cue (e.g., BRUSH—P___), showing cue-independence. Abundant evidence supports this cue-independence property^25,26^. Indeed, whereas direct suppression causes cue-independent forgetting, linking a reminder to novel interfering associations (without attempts to suppress) yields cue-dependent forgetting^29^. Retrieval suppression also impairs performance on both perceptually and conceptually-driven implicit memory tests, consistent with item-level inhibition^14,30–34^.

Although consistent with item-level inhibition, an alternative interference-based hypothesis has been proposed to explain cue-independent forgetting on IP tests. Tomlinson et al. (2009) proposed a two-stage interference model—Search of Associative Memory with Recovery Interference (SAM-RI)—that posits distinct sampling and recovery phases during memory retrieval. In the sampling stage, when seeing a cue (e.g., STATUE) a target is first located (i.e., sampled) in memory, but at this stage is still incomplete (e.g., P__NT), necessitating further recovery. In the recovery stage, the memory’s contents are (attempted to be) recovered and, if successful, a verbal response is generated. The model assumes that, during retrieval suppression, seeing the cue not only samples the original memory, but also updates that trace with a new associated memory based on whatever activity is done during the trial (e.g., the activity of “not thinking about the target”). As a result, this new memory response attached to the target becomes a competing trace that interferes with target recovery. Thus, rather than inhibiting the target, people associate it to an alternate response that is activated whenever the memory is sampled. When a participant receives a semantically related word on an IP test (e.g., BRUSH—P____), the target (e.g., PAINT) may be sampled, but the competing recovery trace (the memory of “not thinking”) causes interference in fully recovering the target^4,35^.

By the foregoing hypothesis, cue-independent forgetting on the IP test should not require that people suppress target retrieval but should happen given any activity that gets associated to the target. To test this, Tomlinson et al. modified the TNT phase to include three tasks. In addition to the conventional Think and No-Think trials, participants were asked to perform trials with a third task that simply required them to press the “Enter” key. For the Press Enter condition, participants quickly pressed the Enter key in response to certain cue words, with no instructions to retrieve or suppress. After the TNT phase, memory was tested for all pairs, first with an SP, and then by an IP test. Tomlinson et al.^4^ replicated the typical TNT results with more forgetting in the No-Think condition compared to the Baseline condition for both SP and IP tests. Interestingly, they also found that the Press Enter condition produced similar results to the No-Think condition on both tests. They argued that forgetting associated with retrieval stopping is not due to inhibition, but rather to recovery interference.

Although these data suggest that suppressing retrieval may not be necessary to induce forgetting, there were unusual features of the experimental design that were not immediately apparent in the main manuscript that recommend caution in reaching this conclusion. The first is that Tomlinson et al.^4^ used a 19-block trial-and-error procedure in the TNT phase. That is, although participants were provided with Think, No-Think, and Press Enter instructions, they were not told which cues required which task; rather, the participants were left to discover what task to do in response to each cue, by trial-and-error because the task was not overtly signaled by a task cue. If, for a given cue word, the participant guessed the task incorrectly (i.e., they retrieved the associate and said it aloud when the trial was meant to involve a button press), they received error feedback after the trial instructing them that they should have pressed the button. Participants thus had to gradually memorize the task-set for each cue in response to error feedback, a process that likely took many trials. Typically, in the TNT paradigm, task instructions are color-coded (e.g., “Think” cue words appear in green, whereas “No-Think” cue words appear in red^5,7,13^) making the desired task fully clear to participants. Another important design feature is the within-subjects manipulation of the three instructions in the TNT phase; having to guess (randomly) what task to do for each cue likely led to a lengthy process of learning the mapping of cues to tasks, given that there are three task-sets to manage.

These design features may have led to forgetting in the Press Enter condition for reasons other than recovery interference. For instance, people may have made task-set mistakes quite often, given that they had to randomly guess what to do. Even when participants learned what to do for each cue, the lack of an overt task cue may have sometimes led to covert mistakes by initially suppressing retrieval of the target on Press Enter trials and vice versa. Thus, an unknown number of Press Enter trials could be No-Think trials, causing inhibition. Moreover, using a within-subjects design also may have prompted task-set carryover effects between trials. For example, intermixing No-Think and Press Enter trials may have rendered the Press Enter and No-Think task-sets more similar, even when participants were performing each task according to expectation. Such task-set blending could have made participants more likely to spontaneously incorporate retrieval stopping as part of the Press Enter task-set, mirroring similar task-set blending that has been observed in research on retrieval-induced forgetting^36^.

It is also possible that the trial-and-error learning procedure would have caused inhibitory forgetting in the Press Enter condition even if the No-Think condition had never been included in the task at all. Forcing participants to guess which task to perform for each retrieval cue could have led participants to spontaneously engage retrieval stopping as a means of minimizing errors, especially in the beginning of the task. For example, suppose a participant mistakenly retrieves a target on a “Press Enter” trial at the beginning of the TNT phase and receives error feedback directing them to “press enter” the next time they see the cue. If participants comply with this feedback and try to remember the right task next time, it may cause them, upon later seeing that cue, to quickly halt the retrieval process; they may quickly recall making a mistake previously and pause to remember the different task that needs to be done. The halting of retrieval to minimize errors may render Press Enter trials similar to No-Think trials, causing target inhibition, a possibility that seems plausible, given that the instruction to press enter implicitly overrules the retrieval response that participants had systematically acquired during the earlier paired-associates training phase.

Another atypical feature of Tomlinson et al.’s^4^ design was the use of a fixed testing order for administering the SP and IP tests, in which the IP test always appeared last. Typically, in TNT studies, both tests are given within-subjects, with their order counterbalanced^6,29,37^. Ordinarily, similar suppression-induced forgetting effects arise regardless of test order. It is unknown, however, what the impact of test order may be on forgetting effects that arise from pressing the Enter key. Pressing Enter may cause forgetting on the IP test because the IP test appeared last, after the SP test, possibly subjecting it to output interference effects unique to “Press Enter” trials. For example, on the SP test, if the “Press Enter” response was covertly retrieved during attempts to recall the target, this retrieval event may have induced forgetting of the target on the later IP test.

The current experiments tested whether simply pressing a button in response to a cue impairs memory, as previously claimed^4^. We sought to replicate the earlier findings and to identify conditions that produce them. A key goal was to reduce the role of putative inhibition processes associated with task-set errors, task-set carryover, and task stopping and to test whether the button-press related forgetting survives. In Experiment 1, we retained Tomlinson et al.’s^4^ trial-and-error task-set learning but eliminated their within-subjects design by separating the No-Think and Button Press conditions into different groups (each combined with a Think and Baseline condition). This design change removes task-set errors (i.e., the confusion of No-Think and Press Enter cues) and task-set carryover (i.e., the incorporation of retrieval suppression into the Button Press task-set) as explanations of forgetting due to button pressing. Thus, finding Button-Press related forgetting in Experiment 1 would argue that although such factors may contribute to Tomlinson et al.’s effects, they are not necessary conditions. The between-subjects design by itself does not eliminate, however, the error minimization hypothesis: without explicit cuing of the task on each trial, participants may often start to retrieve the associate on button press trials, and then stop retrieval when they remember their prior error in response to the cue, a dynamic that should remain in the trial-and-error design, potentially causing inhibition.

In Experiment 2, we eliminated both the within-subjects design and the trial-and-error task learning procedure during the TNT phase. We overtly cued participants on every Think and No-Think *or* Button Press trial about which task they were meant to perform by presenting the cue words in Green and Red, respectively, as is typically done in the TNT task. This combination of a between-subjects design and explicit task cuing should eliminate task-set errors and task-set carryover, and it should greatly reduce incidental retrieval stopping for the Button Press condition, but not for the No-Think condition, in which retrieval stopping is directly signaled by instructions. If pressing a button in response to a cue causes forgetting, as originally claimed, the forgetting effect should survive these changes in experimental design. If, however, retrieval stopping due to error minimization contributes to Button-Press related forgetting, then Experiment 2 should eliminate the effect. If so, such a finding would cast doubt on the idea that recovery interference underlies suppression-induced forgetting, especially if, under identical conditions, No-Think instructions cause the effect.

The current experiments also tested whether the fixed testing order used by Tomlinson et al. might have contributed to the Button Press forgetting effect. We counterbalanced testing order as is standard and included a large enough sample size in each Experiment so that we could examine forgetting separately for cases in which the IP was tested first, uncontaminated by prior SP retrieval. If pressing a button leads to recovery interference, this effect should arise if the IP test is given first. If output interference from the prior SP test caused to the effect, no forgetting should be found when the IP test appears first.

## Experiment 1

### Method

#### Participants

Eighty-four undergraduates of the Erasmus University Rotterdam participated for course credit. Participants were excluded from (further) participation if they had a self-reported diagnosis of attention deficit hyperactivity disorder (ADHD), did not have Dutch as a first language (learned prior to age 5), were color blind, slept less than 5 h the previous night, or if they did not reach the learning criterion of 50% correct on the final learning test (see Think/No-Think procedure). Four participants were excluded; one was diagnosed with ADHD and three did not achieve learning criterion. Our final sample consisted of 80 participants (*M* = 21.48 years, *SD* = 1.79, 28 men, 52 women) equally divided over two groups. Sensitivity analyses for a repeated measures ANOVA (within-between) interaction with as within-subjects factor ‘Intervening Activity’ (Baseline vs. [Sup]press;) and as between-subjects factor ‘Instructional Group’ show that—with our sample—the smallest effect size we could detect was Cohen’s f = 0.159 (partial eta squared = 0.025). The detectable effect size is small to medium, leaning towards small. Sensitivity analyses for specific contrasts (e.g., Baseline vs. Suppress; or Baseline vs. Press Spacebar) show we would be able to detect a Cohen’s *d* of 0.400, which can be quantified as a small-medium effect size. These detectable effect sizes are substantially smaller than those reported by Tomlinson et al. Informed consent was obtained from all participants. This experiment (and the next) was executed in accordance with principles from the Declaration of Helsinki. Both experiments were approved by the Ethics Review Committee of the Department of Psychology, Education, and Child Studies at Erasmus University Rotterdam.

#### Materials and design

The stimuli consisted of 60 critical (and 18 filler) neutral Dutch word pairs of which cue and target were randomly combined (e.g., IVORY—POEM, WALNUT—BLUE). Words were in part drawn from other studies (B. D. Murray, personal communication, March 7, 2012^17^) and were, in part, newly constructed. Additionally, each pair’s target had an independent probe word together with a single letter stem for response on the final test (e.g., RHYME—P____, COLOR—B____) (see Appendix A). All experimental pairs were counterbalanced and rotated through three experimental instructions: Baseline, Respond, and Suppress or Press Spacebar. The Suppress or Press Spacebar instructions were manipulated as a between-subjects factor. We switched to pressing spacebar because this key is easily accessible. Using the spacebar key (compared to the enter key) circumvents participants accidentally pressing any other keys when responding.

Cues, targets and independent probes within these word groups had been rated on valence, arousal, word length, and word frequency, and on association strength from the independent probe to the target^38^. Valence and arousal were rated on a 7-point scale ranging from 1 *very negative/calm* to 7 *very positive/aroused*. Word frequency (reported per million) was taken from the SUBTLEX-NL database as reported in Moors et al.^38^. Association strength from the independent probe to the target was derived from the Dutch word associations database from the Catholic University Leuven. Values are probability estimates that the IP produces the required target item. There were no significant differences between the words groups for any of the variables.

#### Think/no-think procedure

The experiment was run with E-prime 2.0 (Psychology Software Tools, Pittsburgh, PA) on a 1920 × 1200-pixel screen. Throughout the experiment, the experimenter sat behind the participant and scored vocal responses, gave instructions, and provided verbal encouragement when necessary.

##### Study and learning phase

For ease of learning, pairs were divided over three sets and participants learned one set before moving on to the next. Participants first studied a set of 20 critical pairs and 6 filler pairs which were individually presented in white on a black background in the middle of the screen for 4000 ms (400 ms ITI). A pseudo-randomized test-feedback cycle followed in which participants responded with the target into a microphone when a cue appeared. The cue remained on the screen for 3500 ms or until the participant responded. Each trial concluded with a display of the correct target in violet for 1000 ms (400 ms ITI) regardless of the answer provided. Participants needed to achieve a 50% correct criterion for critical words (i.e., 10 words) to continue to the second set of 26 words. If the participant failed to reach this criterion, the test-feedback cycle was repeated once. Regardless of the percentage correct (on set repetition) the participant continued with the second and third set following an identical procedure (if necessary, with a repetition). Participants concluded this phase with a learning test covering words from the three sets without receiving trial feedback. Again, they were required to reach the 50% learning criterion. If the participant failed to reach criterion, the complete study and learning phase was repeated once. Any participant who recalled less than 50% of the word pairs correctly on the second learning test was excluded from further participation.

##### Think/no-think phase

During this phase participants received a combination of either two types of trials: Respond and Suppress or Respond and Press Spacebar (i.e., between group manipulation). Baseline items were not presented in this phase. All trials started with a 400 ms fixation cross, displayed possible feedback in violet for 500 ms, and ended with a 400 ms ITI. On Respond trials, participants were instructed to say the correct target word as quickly as possible into the microphone. The cue word was displayed on the screen for 4000 ms or until the subject gave an answer. A Respond trial always concluded with a feedback display of the correct target. On Suppress trials, participants were instructed to focus their attention on the cue for 3000 ms, whilst avoiding retrieval of the associated response word. If participants accidentally spoke an answer on these trials, they were provided with the feedback ‘INCORRECT!’ displayed on screen accompanied by a loud beep. In the group that received Press Spacebar instructions, participants were required on Press Spacebar trials to press the spacebar within 1500 ms after the cue word appeared on screen. If the spacebar was pressed in a timely way, no feedback was displayed on screen. If the participant failed to press within the given time, the feedback ‘FASTER!’ was presented on screen accompanied by a loud beep. How participants needed to respond to each item was not overtly signaled; rather, participants had to discover what to do in response to each cue, by trial-and-error, as was done in Tomlinson et al.^4^.

Participants started the TNT phase with 36 practice fillers trials (18 Respond, 18 Suppress or Press Spacebar), followed by the experimental TNT trials. The TNT phase consisted of six blocks each with 96 trials, half of which were Respond cues and half of which were Suppress or Press Spacebar cues. Items were presented in a pseudo-randomized order, with each cue appearing twice in a block and with no more than three items in the same condition appearing in a row. For a given participant, repetitions of an item always were presented in the same condition. Overall, each cue was repeated 12 times in the TNT phase and the six blocks were separated by 30–45 s breaks.

##### Final test phase

All 60 critical pairs were tested with a SP test using the original cue (e.g., IVORY—____) and an IP test using a word associated with the target together with the first letter of the target (e.g., RHYME—P____). Within each test, cues appeared once in white font in the middle of the screen for 10 s (400 ITI) and participants were instructed to say the correct target into the microphone for all items regardless of instructions in the TNT phase. The item disappeared from screen ahead of time when an answer was given. The order of the SP and IP tests was counterbalanced across participants, and the average serial position within each test type for all item types (Baseline, Respond, and Suppress or Press Spacebar) was carefully matched.

#### Statistical analyses

All data were analyzed with JASP (2022, version 0.16.3)^39^. JASP is a freely accessible, open-source package for classical (i.e., Null Hypothesis Significance Testing) and Bayesian statistical analyses. For analyses we only used the pairs for which participants were able to recall the target on the final learning test^5,17^. Though we were specifically interested in forgetting effects (i.e., Baseline vs. Suppress; Baseline vs. Press Spacebar) we also report facilitation effects (i.e., Baseline vs. Respond); we report these effects in separate analyses. This is a conventional analysis method because suppression induced forgetting (i.e., below-baseline forgetting for No-Think items) and retrieval effects (i.e., above-baseline remembering for Think items) are thought of as separate theoretical entities^15,21,40–42^. In all analyses, Intervening Activity (e.g., Baseline vs. Suppress; or Baseline vs. Press Spacebar; or Baseline vs. Respond) was analyzed as a within-subjects factor. Instruction was manipulated between-subjects, and this determined whether participants needed to Suppress *or* Press Spacebar during the TNT phase (i.e., the factor Instructional Group). Both test types (i.e., SP and IP test data) entered into the main analysis. Lastly, item counterbalancing and test counterbalancing were included as factors to account for item effects and test order effects. In case of violations of assumptions, we report appropriate corrections.

We report analyses of variance (ANOVAs) to examine the after-effects of Suppress and Press Spacebar instructions on the final test. For critical non-significant effects we also report an exclusion Bayes Factor (BF_exclusion_, matched across models) to quantify evidence for the null effect. For instance, A BF_exclusion_ = 5 means that data are 5 times more likely under the models that exclude that specific effect than under the models with this effect. Note that for Bayesian analyses item counterbalancing was left out as a factor in all analyses because estimating all possible models (when including all five fixed factors) simply exceeded the computational power available (i.e., > 2 billion models needed to be estimated).

Data and associated analyses for this experiment have been made publicly available via the Open Science Framework and can be accessed at https://osf.io/e5myk/.

## Results

### Learning phase

Learning test performance was sufficiently high; participants recalled 69.1% (*SD* = 0.11) of the critical pairs on the criterion test at the end of learning. Crucially, there was no difference in learning test performance between the Instructional Groups, *F*(1, 60) = 0.088, *p* = 0.767, η_p_^2^ = 0.001. Nor was there a difference between word groups that were to be assigned to the different intervening activities in the TNT phase (i.e., Respond, Baseline, and Suppress/Press Spacebar), *F*(2, 120) = 0.348, *p* = 0.707, η_p_^2^ = 0.006. There was no Intervening Activity × Instructional Group interaction in learning performance, *F*(2, 120) = 0.083, *p* = 0.921, η_p_^2^ = 0.001. Overall, this shows that the Suppress and Press Spacebar groups entered the TNT phase with comparable levels of learning.

### Final recall performance

#### Suppression effects

**Figure 1:**
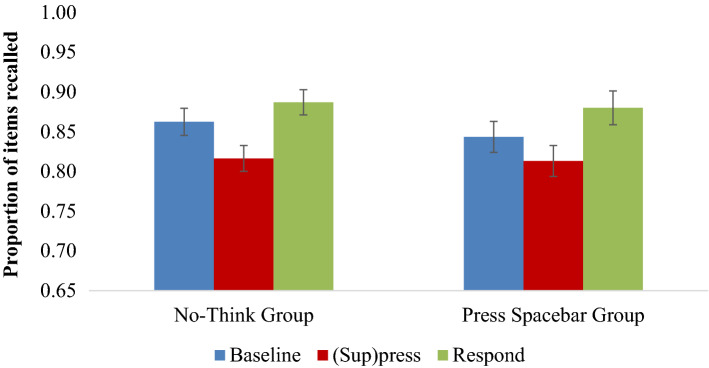
Mean recall proportion for Baseline (blue), (Sup)press (red), and Respond (green) aggregated by Test Type for Experiment 1. Error bars represent standard errors of the mean.

Replicating Tomlinson et al.’s findings, we observed below-baseline forgetting, *F*(1, 60) = 11.963, *p* = 0.001, η_p_^2^ = 0.166, regardless of whether participants suppressed the target or pressed spacebar in response to seeing the cue, *F*(1, 60) = 0.510, *p* = 0.478, η_p_^2^ = 0.008. Indeed, the absence of this interaction was confirmed by a complementary Bayesian analysis that shows the null hypothesis is supported, BF_exclusion_ = 4.18 (see Fig. 1). There was no reliable evidence that this effect varied with Test type* F*(1, 60) = 2.564, *p* = 0.115, η_p_^2^ = 0.041. The interactions of Test type with Activity and Test type with Instructional Group were not significant, *F*s ≤ 2.684, *p*s ≥ 0.107.

We observed that overall more items were recalled on the SP test than on the IP test, *F*(1, 60) = 131.171, *p* < 0.001, η_p_^2^ = 0.686. This is a common effect in TNT studies as the SP cue is familiar and specifically trained, whereas the IP cue is novel^3^. The IP test is of particular significance because an independent, semantically related cue is used to probe memory and any forgetting is often taken to reflect active inhibition^27^. Tomlinson et al.^4^ specifically showed that cue-independent forgetting of this sort not only arose from retrieval suppression, but also arose when participants pressed a button in response to the cue. To test whether we could reproduce these key independent probe results, we separately analyzed the IP data (The SP data were not analyzed separately because of ceiling effects; we present data distributions and full table of means for the SP test data in Appendix B). On the IP test, we observed overall below-baseline forgetting, *F*(1, 60) = 11.113, *p* = 0.001, η_p_^2^ = 0.156, that did not interact with whether participants suppressed the target or pressed the spacebar, *F*(1, 60) = 0.404, *p* = 0.528, η_p_^2^ = 0.007. Here too, a complementary Bayesian analysis shows the null hypothesis is supported and that the evidence is in favor of the absence of an interaction, BF_exclusion_ = 3.80. Thus, pressing the spacebar induced forgetting on a test that theoretically should measure memory inhibition.

#### Facilitation effects

Facilitation effects were clearly observable as evidenced by higher recall of Respond items compared to Baseline items on the final test,* F*(1, 60) = 8.399, *p* = 0.005, η_p_^2^ = 0.123. This effect did not interact with whether Suppress or Press Spacebar instructions were administered, *F*(1, 60) = 0.322, *p* = 0.573, η_p_^2^ = 0.005. Besides a main effect of Test Type,* F*(1, 60) = 192.137, *p* < 0.001, η_p_^2^ = 0.762, none of the other main effects or interactions of Intervening Activity, Instructional Group, or Test Type were significant, *F*s ≤ 2.548, *p*s ≥ 0.116. Hence, overall facilitation was present in the data and this was unaffected by whether participants were instructed to suppress or press the spacebar.

## Experiment 2

### Method

Experiment 2 was identical to Experiment 1 except that the instructions for items in the TNT phase were color coded: Respond trials were displayed in green font and Suppress trials or Press Spacebar trials were presented in red. Eighty-one undergraduates from the Erasmus University Rotterdam participated for course credit. Identical exclusion criteria were used as in Experiment 1. Only one participant was excluded for failing to achieve criterion within two repetitions. The final sample consisted of 80 participants (*M* = 20.68 years, *SD* = 2.50, 11 men; 69 women) equally divided over both groups.

## Results

### Learning phase

On the learning test, participants recalled 68.7% (*SD* = 0.11) of critical pairs. Importantly, the groups that received Suppress or Press Spacebar instructions recalled comparable numbers of critical pairs, *F*(1, 60) = 1.435, *p* = 0.236, η_p_^2^ = 0.023. Moreover, there were no differences between word groups that were to be assigned to different conditions in the TNT phase, *F*(2, 120) = 0.212, *p* = 0.809, η_p_^2^ = 0.004 and there was no Intervening Activity × Instructional Group interaction, *F*(2, 120) = 0.534, *p* = 0.588, η_p_^2^ = 0.009.

### Final recall performance

#### Suppression effects

**Figure 2:**
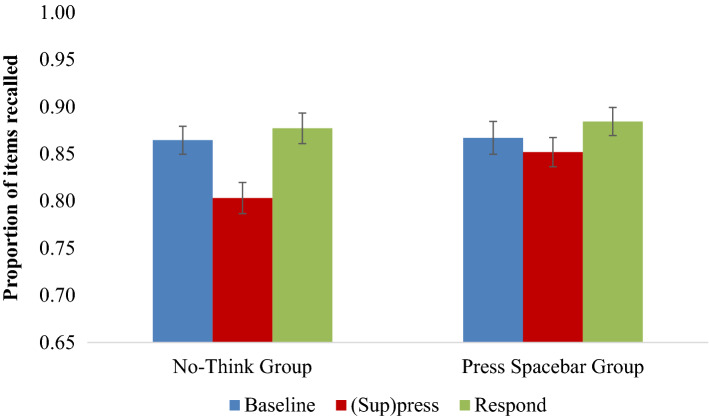
Mean proportion of recall for Baseline (blue), (Sup)press (red), and Respond (green) aggregated by Test Type for Experiment 2. Error bars represent standard errors of the mean.

We expected that removing the trial-and-error learning procedure during the TNT phase by providing participants with color-coded task cues would abolish the forgetting effects for the Press Spacebar group. Across both groups, we observed an overall below-baseline forgetting effect, *F*(1, 60) = 11.876, *p* = 0.001, η_p_^2^ = 0.165 but this effect was qualified by an Intervening Activity × Instructional Group interaction, *F*(1, 60) = 4.321, *p* = 0.042, η_p_^2^ = 0.067 that did not vary by Test type, *F*(1, 60) = 1.996, *p* = 0.163, η_p_^2^ = 0.032. This interaction shows that below-baseline forgetting significantly varied depending on whether participants were told to suppress retrieval or to press the spacebar (see Fig. 2). Follow-up analyses confirm that below-baseline forgetting occurred only in the group that suppressed retrieval, *F*(1, 30) = 13.134, *p* = 0.001, η_p_^2^ = 0.304, but not in the group that pressed the spacebar, *F*(1, 30) = 1.116, *p* = 0.299, η_p_^2^ = 0.036. Because the lack of effect in the Press Spacebar group is theoretically important, we further examined whether this effect is truly supportive of the null by performing an additional Bayesian analysis for this specific comparison. Indeed, this analysis confirms the null effect is supported, BF_exclusion_ = 3.18.

Again, more items overall were recalled on the SP test than on the IP test, *F*(1, 60) = 191.524, *p* < 0.001, η_p_^2^ = 0.761. SP test performance was affected by ceiling effects and therefore was not analyzed further (the data distributions and a table of means are presented in Appendix C). Because of the a priori theoretical importance of the IP data for showing the involvement of inhibitory processes, we performed focused analyses of the IP data. In line with the foregoing analysis on the aggregated SP/IP data, we found overall below-baseline forgetting, *F*(1, 60) = 7.870, *p* = 0.007, η_p_^2^ = 0.116. Below-baseline forgetting on the IP test, however, was affected by whether participants suppressed items or instead pressed a spacebar, *F*(1, 60) = 4.288, *p* = 0.043, η_p_^2^ = 0.067. Consistent with our hypothesis, these data confirm that forgetting was specific to the Suppress group, *F*(1, 30) = 9.956, *p* = 0.004, η_p_^2^ = 0.249, and that it did not occur for the Press Spacebar group, *F*(1, 30) = 0.335, *p* = 0.567, η_p_^2^ = 0.011. Again, Bayesian analyses confirm this finding represents evidence supporting the null, BF_exclusion_ = 3.73. This finding shows that providing participants with color-coded cues abolishes the forgetting effect for pressing spacebar, whilst preserving it in the retrieval suppression group.

#### Facilitation effects

Most of the main effects or interaction effects of Intervening Activity, Instructional Group, and Test Type were not significant, *F*s ≤ 1.951, *p*s ≥ 0.168, besides Test type, *F*(1, 60) = 327.878, *p* < 0.001, η_p_^2^ = 0.845 and a Test type × Intervening Activity interaction, *F*(1, 60) = 13.545, *p* < 0.001, η_p_^2^ = 0.184, showing that recall was highest for the SP data and specifically for respond items tested on the SP test. This two-way interaction varied with Instructional Group, *F*(1, 60) = 5.462, *p* = 0.023, η_p_^2^ = 0.083, which was driven by Group differences on the SP test, *F*(1, 60) = 3.875, *p* = 0.054, η_p_^2^ = 0.061, and not the IP test, *F*(1, 60) = 1.748, *p* = 0.191, η_p_^2^ = 0.028.

## Combined analyses of experiments 1 and 2

To investigate potential contributions of output interference caused by placing the SP test before the IP test on the suppression effects (as was done by Tomlinson et al.^4^), we combined the data from both experiments and examined forgetting effects separately for cases in which the IP was tested first (i.e., uncontaminated by prior SP retrieval) and tested second (i.e., potentially contaminated by prior SP retrieval). If pressing a spacebar causes recovery interference, below-baseline forgetting should clearly arise if the IP test is given first. However, if the forgetting effect for the Press Spacebar group reflects output interference from the prior retrieval of SP items, then it should materialize only when the IP test is given second. For these analyses ‘Experiment’ was added as an additional factor.

When the IP test was tested first, we observed overall below-baseline forgetting, *F*(1, 60) = 9.870, *p* = 0.003, η_p_^2^ = 0.141, which interacted with Instructional Group, *F*(1, 60) = 4.430, *p* = 0.040, η_p_^2^ = 0.069. Breaking this interaction down, whereas significant forgetting (Baseline vs. Suppress) was observed when participants performed retrieval suppression, *F*(1, 30) = 12.569,* p* = 0.001, η_p_^2^ = 0.295, no reliable forgetting (Baseline vs. Press Spacebar) was found when participants instead pressed spacebar, *F*(1, 30) = 0.594, *p* = 0.447, η_p_^2^ = 0.019. Crucially, the evidence is in favor of the null effect, BF_exclusion_ = 3.77, showing that forgetting in the Press Spacebar group was indeed absent. Thus, considering all the available data, there is not a reliable effect of pressing the spacebar on forgetting when output interference is properly controlled, unlike in the retrieval suppression condition. In contrast, when the IP test appeared after the SP test, as it did in Tomlinson et al.’s^4^ experiment, there was a significant effect of forgetting in the Press Spacebar condition, *F*(1, 30) = 6.817,* p* = 0.014, η_p_^2^ = 0.185, consistent with the possibility that the spacebar-induced forgetting effect arose in part from output interference.

**Figure 3:**
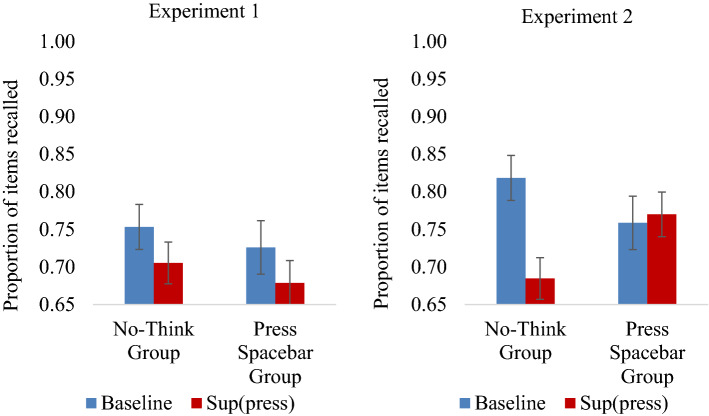
Mean proportion recalled on the Independent Probe test when it appeared before the Same Probe test, for Baseline (blue) and (Sup)press (red) split out by Experiment: Experiment 1 (left panel) and Experiment 2 (right panel). Error bars represent standard errors of the mean.

Is the spacebar induced forgetting effect entirely caused by output interference? We considered whether multiple mechanisms contribute to this effect, including both inhibition and output interference. Specifically, we hypothesized that inhibition may play a greater role in the Press Spacebar condition in Experiment 1, given the trial-and-error training procedure used in the TNT phase of that study, and the potential for spontaneous retrieval stopping to contribute to error minimization. If so, we should find greater evidence for forgetting when the IP test appears first in Experiment 1, but not in Experiment 2. Supporting this hypothesis, the forgetting effect was reliable in Experiment 1, *F*(1, 30) = 5.431,* p* = 0.027, η_p_^2^ = 0.153, and did not interact with Instructional group *F*(1, 30) < 0.001,* p* = 0.985, η_p_^2^ < 0.001. Bayesian analysis shows that the absence of an interaction was over 2.5 times more likely than the presence of this particular interaction, BF_exclusion_ = 2.66. Together the analyses show that there was below-baseline forgetting for the Press Spacebar and No-Think Groups in Experiment 1. In contrast, in Experiment 2, below-baseline forgetting, *F*(1, 30) = 4.790,* p* = 0.037, η_p_^2^ = 0.138, did interact with Group, *F*(1, 30) = 6.717,* p* = 0.015, η_p_^2^ = 0.183. Break-down of this effect shows that below-baseline forgetting did not occur in the Press Spacebar Group, *F*(1, 15) = 0.0.102,* p* = 0.754, η_p_^2^ = 0.007 and was limited to the No-Think Group, *F*(1, 15) = 0.9.511,* p* = 0.008, η_p_^2^ = 0.388. Indeed, the absence of below-baseline forgetting in the Press Spacebar group was further confirmed by a substantial Bayes Factor, BF_exclusion_ = 3.16, showing that this is a true null effect. These findings suggest that the effect in Experiment 1 was not solely caused by output interference. Confirming this difference, when the IP test appeared first, the two-way interaction of Instructional Group and Forgetting (Baseline vs. Suppress/Press Spacebar) varied with Experiment,* F*(1, 60) = 4.337, *p* = 0.042, η_p_^2^ = 0.067 (see Fig. 3).

## Discussion

Three main findings emerge from the current work. First, we replicated the effect whereby pressing a button in response to a cue induces below-baseline forgetting on an independent probe test, even when no overt suppression instructions were given. Indeed, we generalized this finding to a design in which we separated the Suppress and Press Spacebar conditions into distinct groups. This generalization shows that forgetting due to button pressing does not require the intermixing of Suppress and Press Spacebar items in the TNT phase, as was done by Tomlinson et al.^4^, arguing that task-set carryover and task-set errors are not necessary to produce the forgetting effect. This does not imply, however, that these factors did not also contribute to the spacebar-induced forgetting effect in the original experiment^4^, but merely indicates that they are not necessary causes.

Second, we found that we could fully abolish the below-baseline forgetting effect caused by pressing the spacebar by both using a between-subjects design and eliminating the trial-and-error procedure that was used in the TNT phase by Tomlinson et al.^4^. In Tomlinson et al.’s procedure, participants were required to discover which task they needed to perform for each cue in the TNT phase. We instead followed the typical design of TNT studies^5,19,32,37^ by clearly indicating what task needed to be done by the color of the cue (i.e., green for Respond; red for Suppress or Press Spacebar). When the task was clearly cued and Suppress and Press Spacebar trials were done in separate groups, no below-baseline forgetting remained for the Press Spacebar condition. In contrast, under precisely the same conditions, suppression led to forgetting on the final test. This pattern clearly shows that forgetting in Tomlinson et al.^4^ derived in part from the unusual trial-and-error learning procedure they used in their TNT phase, and not simply from pressing a key in response to the cue.

Third, we found that the spacebar-induced forgetting effect on our IP test also is partially produced by the biased output order Tomlinson et al. used on their final test. To test this, we analyzed IP test data that were uncontaminated by the prior recall of items on the SP test (i.e., when the IP was given first) and that thus provide a purer test of cue-independent forgetting. In these analyses, we found that below-baseline forgetting always occurred (i.e., in both experiments) when people suppressed retrieval. In contrast, when the candidate sources of inhibition that we hypothesized (task-set errors, task-set carryover, and retrieval stopping do minimize errors) were fully controlled (Experiment 2), there was no trace of forgetting for the Press Spacebar group. This finding is significant because in the original study^4^, the SP test always appeared before the IP test, confounding test type with test position. Indeed, in our experiments, when the SP test was given before the IP test, replicating this confound, forgetting on the IP test indeed occurred for the Press Spacebar group, suggesting that the preceding SP test may have been important in causing Tomlinson et al.’s reported effects.

Taken together, these findings indicate that simply pressing spacebar in response to a cue is not enough to induce forgetting, contrary to prior conclusions. If this had been true, spacebar-induced forgetting should have occurred regardless of the elimination of the within-subjects design (Experiment 1) and the trial-and-error learning procedure (Experiment 2). Instead, we found that the removal of the trial-and-error learning (in concert with the use of a between-subjects design) eliminated the spacebar-induced forgetting effect. Thus, whereas Tomlinson et al.’s^4^ procedure yields reproducible forgetting, it does so for reasons other than the mere association of a motor response to a memory. This raises the question of what mechanism might produce this effect.

At least two mechanisms may contribute to our findings. First, our data are consistent with a role of retrieval stopping in minimizing task-set errors during Tomlinson et al.’s trial-and error learning procedure in the TNT phase. By this hypothesis, the initial training of cue-target pairs induces a strong tendency to retrieve a target when its cue is presented, a task-set that is initially associated to every retrieval cue in the experiment. Later, when participants are given the same cues during the TNT task, this training likely initiates a retrieval response automatically, by default. In Tomlinson et al.’s trial-and-error learning procedure, many trials were likely needed for participants to successfully associate the No-Think and Button Press instruction to each individual cue word, likely with many “misfires” of the retrieval task-set. With many repetitions of a given item, however, participants would come to retrieve the new Button Pressing instruction more quickly, allowing it to supplant the habitual retrieval response and produce the desired button pressing behavior. Even when button pressing behavior replaced retrieval overtly, effort may have been required to cancel retrieval for a given cue to avoid doing the wrong task, perhaps leading participants to pause any response to a cue until the proper instruction was identified. This retrieval-pausing may have engaged a retrieval stopping mechanism that led to inhibition. Thus, spacebar-induced forgetting would not result from associating a motor response to a memory; rather, forgetting reflected the need to interrupt retrieval, inducing unintended suppression-induced forgetting. This mechanism accounts for the button-press-induced forgetting in Experiment 1, especially when the IP test appeared first. It also can explain why forgetting did not occur in Experiment 2 (especially when IP occurred first): if providing color-coded task cues increased the speed and accuracy of task-set selection on button press trials, it should have reduced any chance of committing a task-set error, reducing the need for retrieval stopping.

The second mechanism likely to contribute to spacebar-induced forgetting is a form of output interference induced by prior retrieval on the SP test. According to this hypothesis, learning to press a spacebar upon seeing a retrieval cue associates that cue with a novel button pressing response. Later, when that cue appears on the final SP memory test, it elicits, in parallel, retrieval of both the originally trained associate and the button press response, which compete for control of behavior. Quite often, participants may covertly retrieve the button press response on such a test but withhold it because they are aware that no button presses are required on the final test. However, the covert retrieval of the button press response on the SP test may lead to forgetting on the later IP test of the same target item, perhaps due to retrieval-induced forgetting effects, an effect that would not occur for Baseline items that were never associated to button press responses. Such an interference effect is compatible with our testing order effects in the Press Spacebar condition across Experiments 1 and 2, and the apparent absence of such effects for the retrieval-suppression condition, for which button presses were never associated to the No-Think cues. Notably, this mechanism is qualitatively different from the recovery interference hypothesis, according to which the button press response is associated to the target itself, not the cue, and which causes forgetting by interference, not inhibition.

Taken together, these findings indicate that, while button-press-induced forgetting can be replicated, any lasting forgetting effect produced by this manipulation does not arise from pressing the spacebar per se; rather, it arises as an artifact of the unusual trial-and-error task learning procedure adopted by Tomlinson et al.^4^. This method introduces unintended incentives for participants to suppress retrieval, in part, because they need to minimize errors during task-set selection. When this trial-and-error factor is controlled, button-press induced forgetting disappears overall, although a residual output interference component occurs. In contrast, when we controlled for trial-and error learning and eliminated output interference as a potential source of forgetting, suppression-induced forgetting remained highly robust. Thus, under identical test conditions (color coded instruction cues, matched output order), forgetting occurred after suppression, but not after pressing buttons. Collectively, the current findings cast doubt on the viability of recovery interference as an explanation for suppression-induced forgetting in the TNT paradigm. They reinforce, however, the hypothesized role of inhibitory processes during retrieval suppression, consistent with cognitive and neural evidence for the role of inhibitory control in suppression-induced forgetting^2,5,6,22,26,32^.

## Supplementary Information

Supplementary Information.

## Data Availability

The data are available on the Open Science Framework in JASP files and CSV files https://osf.io/e5myk/.
