# Supplementary material for: On the role of inhibition in suppression-induced forgetting
Source: Sci Rep. 2023 Mar 14;13:4242. doi: 10.1038/s41598-023-31063-3 (PMC10015003; doi:10.1038/s41598-023-31063-3)
Supplement: Supplementary file 1 — Supplementary Information. [file 41598_2023_31063_MOESM1_ESM.docx]

**Appendix A**

**Table A1**

*Cue, target, and independent probe for experimental and filler pairs.*

| **Dutch Experimental Pairs** | | | |  | **English Translations** | | | |
| --- | --- | --- | --- | --- | --- | --- | --- | --- |
| **Cue** | **Target** | | **Independent Probe** |  | **Cue** | | **Target** | **Independent Probe** |
| GELEI | RUW | | GROF – R |  | JELLY | | CRUDE | COARSE |
| JURK | VAANDEL | | VLAG – V |  | DRESS | | BANNER | FLAG |
| VORK | PAARD | | RUITER – P |  | FORK | | HORSE | RIDER |
| MOLEN | VIN | | VIS – V |  | MILL | | FIN | FISH |
| ZUIL | VIOOL | | SNAAR – V |  | COLUMN | | VIOLIN | STRING |
| AZIE | RIVIER | | STROOM – R |  | ASIA | | RIVER | STREAM |
| SPROET | BOODSCHAP | | SMS – B |  | FRECKLE | | MESSAGE | SMS |
| KONING | HEUVEL | | BERG – H |  | KING | | HILL | MOUNTAIN |
| HOOI | PERRON | | TREIN – P |  | HAY | | PLATFORM | TRAIN |
| GORDIJN | POSTER | | MUUR – P |  | CURTAIN | | POSTER | WALL |
| RADIO | SNEEUW | | WINTER – S |  | RADIO | | SNOW | WINTER |
| STOEL | VUUR | | VLAM – V |  | SEAT | | FIRE | FLAME |
| SPOOK | NEEF | | TANTE – N |  | GHOST | | NEPHEW | AUNT |
| BONT | FORNUIS | | KOKEN – F |  | FUR | | STOVE | TO COOK |
| HELM | CASSETTE | | VIDEO – C |  | HELMET | | CASSETTE | VIDEO |
| STANDBEELD | VERF | | KWAST – V |  | STATUE | | PAINT | BRUSH |
| KIN | GRAS | | TUIN – G |  | CHIN | | GRASS | GARDEN |
| VAT | NON | | KLOOSTER – N |  | BARREL | | NUN | CLOISTER |
| TOMAAT | VEST | | KNOPEN – V |  | TOMATO | | VEST | BUTTONS |
| UUR | CENT | | MUNT – C |  | HOUR | | CENT | COIN |
| BLOUSE | ZWAARD | | RIDDER – Z |  | BLOUSE | | SWORD | KNIGHT |
| SMARAGD | BALLET | | DANS – B |  | EMERALD | | BALLET | DANCE |
| MUSEUM | KLUIS | | CODE – K |  | MUSEUM | | VAULT | CODE |
| KORST | LERAAR | | LEERLING – L |  | CRUST | | TEACHER | PUPIL |
| BEZEM | POP | | SPEELGOED – P |  | BROOM | | DOLL | TOYS |
| KAAK | NOORD | | ZUID – N |  | JAW | | NORTH | SOUTH |
| TOLK | CIRKEL | | KRING – C |  | INTERPRETER | | CIRCLE | CIRCLE |
| NONCHALANT | BOEK | | LEZEN – B |  | NONCHALANT | | BOOK | TO READ |
| RAAM | MAAG | | ORGAAN – M |  | WINDOW | | STOMACH | ORGAN |
| UIL | KANTOOR | | BUREAU – K |  | OWL | | OFFICE | DESK |
| PATENT | EI | | DOOIER – E |  | PATENT | | EGG | YOLK |
| KRANT | PLANEET | | AARDE – P |  | NEWSPAPER | | PLANET | EARTH |
| CLOWN | LENS | | BRIL – L |  | CLOWN | | LENS | GLASSES |
| GEWOONTE | TOREN | | KERK – T |  | HABIT | | TOWER | CHURCH |
| GANG | KETEL | | STOOM – K |  | CORRIDOR | | KETTLE | STEAM |
| MACHINE | DEUGD | | GEDULD – D |  | MACHINE | | VIRTUE | PATIENCE |
| STRIK | LAARS | | SCHOEN – L |  | BOW | | BOOT | SHOE |
| DORP | OLIJF | | OLIE – O |  | VILLAGE | | OLIVE | OIL |
| HOEK | ZALF | | TUBE – Z |  | CORNER | | OINTMENT | TUBE |
| SPIEREN | DWERG | | KLEIN – D |  | MUSCLES | | DWARF | SMALL |
| DUN | METER | | LENGTEMAAT – M |  | THIN | | METER | LENGTH |
| ITEM | SLAPEN | | DROOM – S |  | ITEM | | TO SLEEP | DREAM |
| VLECHT | VALK | | ROOFVOGEL – V |  | BRAID | | FALCON | BIRD OF PREY |
| KWART | ARM | | ELLEBOOG – A |  | QUART | | ARM | ELBOW |
| NEDERIG | LAMP | | LICHT – L |  | HUMBLE | | LAMP | LIGHT |
| VOETBAL | TIENER | | JONG – T |  | SOCCER | | TEENAGER | YOUNG |
| STAD | FLUIT | | ORKEST – F |  | CITY | | FLUTE | ORCHESTRA |
| WALNOOT | BLAUW | | KLEUR – B |  | WALNUT | | BLUE | COLOR |
| IVOOR | GEDICHT | | RIJM – G |  | IVORY | | POEM | RHYME |
| ROOM | CITROEN | | ZUUR – C |  | CREAM | | LEMON | SOUR |
| LAAN | KAST | | LADE – K |  | LANE | | CABINET | DRAWER |
| HARDLOPER | NACHT | | DONKER – N |  | RUNNER | | NIGHT | DARK |
| RUM | REPARATIE | | GARAGE – R |  | RUM | | REPAIR | GARAGE |
| HEK | MOS | | BOS – M |  | FENCE | | MOSS | FOREST |
| SPRONG | HUIS | | BAKSTEEN – H |  | LEAP | | HOUSE | BRICK |
| SCHOOL | PAPIER | | PEN – P |  | SCHOOL | | PAPER | PEN |
| SCHADUW | WORTEL | | KONIJN – W |  | SHADOW | | CARROT | RABBIT |
| SCHORT | VAKANTIE | | ZON – V |  | APRON | | HOLIDAY | SUN |
| BUS | KELDER | | ZOLDER – K |  | BUS | | CELLAR | ATTIC |
| PRIESTER | SCHIP | | KAPITEIN – S |  | PRIEST | | SHIP | CAPTAIN |
|  |  | |  |  |  | |  |  |
| **Dutch Filler Pairs** | | | |  | **English Translations** | | | |
| **Cue** | | **Target** | **Independent Probe** |  | **Cue** | **Target** | | **Independent Probe** |
| KINDERKAMER | | MAND | RIET – M |  | NURSERY | BASKET | | REED |
| GLOBE | | KLOK | TIJD – K |  | SPHERE | CLOCK | | TIME |
| WERKTUIG | | VINGER | DUIM – V |  | UTENSIL | FINGER | | THUMB |
| GEREEDSCHAP | | SCHRIJVER | AUTEUR – S |  | TOOL | WRITER | | AUTHOR |
| PARAPLU | | SCHAAR | KNIPPEN – S |  | UMBRELLA | SCISSORS | | TO CUT |
| GOLFER | | TAXI | CHAUFFEUR – T |  | GOLFER | TAXI | | DRIVER |
| LIPGLOSS | | DEUR |  |  | LIPGLOSS | DOOR | |  |
| GEBRUIK | | VIERKANT |  |  | CUSTOM | SQUARE | |  |
| LAKEN | | DOKTER |  |  | SHEETS | DOCTOR | |  |
| ONDERDEEL | | KOM |  |  | PART | BOWL | |  |
| APPARAAT | | KOEIEN |  |  | APPLIANCE | COWS | |  |
| SPRAY | | SNOER |  |  | SPRAY | CORD | |  |
| FORMULIER | | TIJM |  |  | FORM | THYME | |  |
| VERREKIJKER | | KAUWGOM |  |  | BINOCULARS | GUM | |  |
| SALADE | | KOFFIE |  |  | SALAD | COFFEE | |  |
| WOLK | | STRAAT |  |  | CLOUD | STREET | |  |
| RECLAME | | BRUG |  |  | COMMERCIAL | BRIDGE | |  |
| BADKAMER | | IJZER |  |  | BATHROOM | IRON | |  |

*Note*: The original materials were in Dutch.

**Appendix B**

**Figure B1**

*Mean Same Probe Test recall proportion for Experiment 1. Boxplots and individual data points (circles) for the No-Think group (top panel) and Press Spacebar group (bottom panel) for Baseline, (Sup)press, and Respond are displayed.*

*
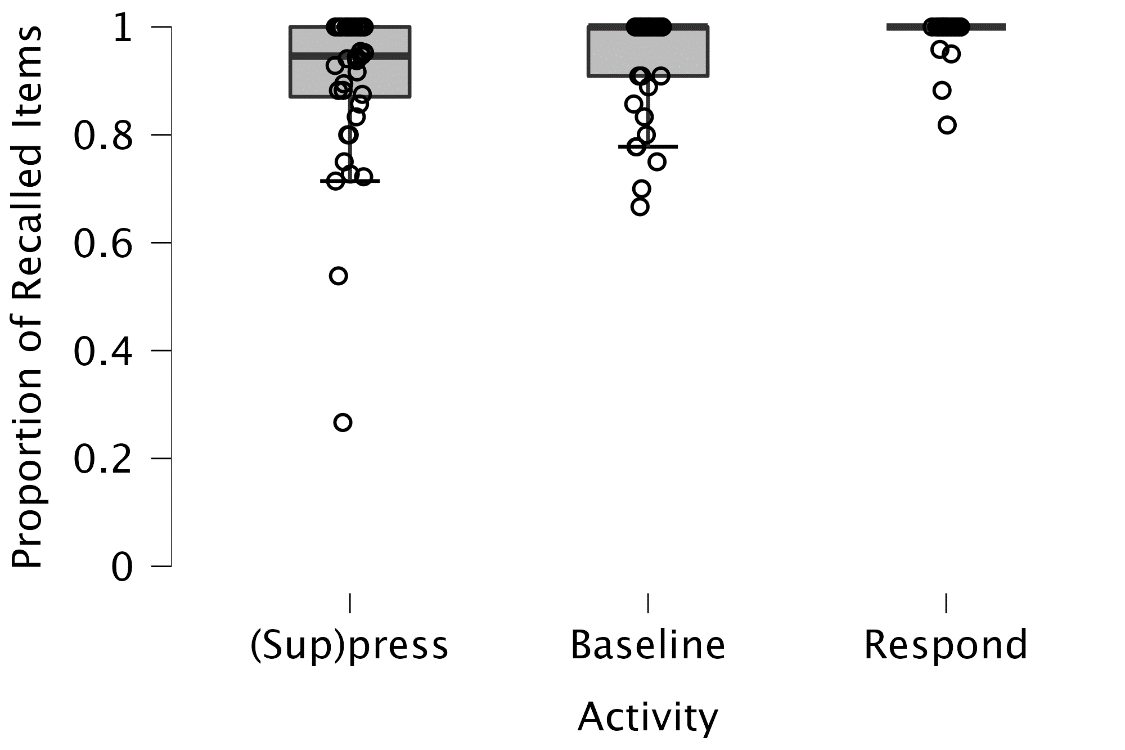
*

*
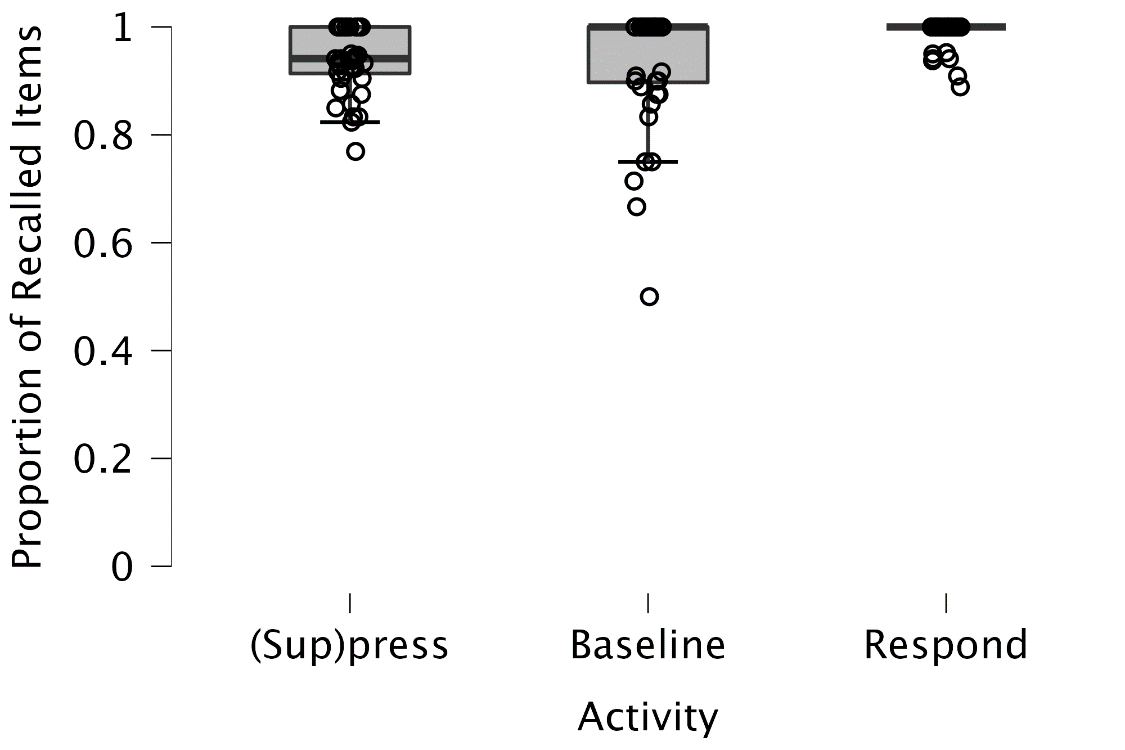
*

Table B2

*Mean (Standard Error) Same Probe Test recall proportion for Experiment 1 for the No-Think group and Press Spacebar group for Baseline, (Sup)press, and Respond*

|  |  | Condition | | |
| --- | --- | --- | --- | --- |
|  |  | (Sup)press | Baseline | Respond |
| Group | No-Think | 0.899 *(0.023)* | 0.944 *(0.015)* | 0.990 *(0.006)* |
|  | Press Spacebar | 0.939 *(0.010)* | 0.931 *(0.018)* | 0.986 *(0.005)* |

**Appendix C**

**Figure C1**

*Mean Same Probe Test recall proportion for Experiment 2. Boxplots and individual data points (circles) for the No-Think group (top panel) and Press Spacebar group (bottom panel) for Baseline, (Sup)press, and Respond are displayed.*

*
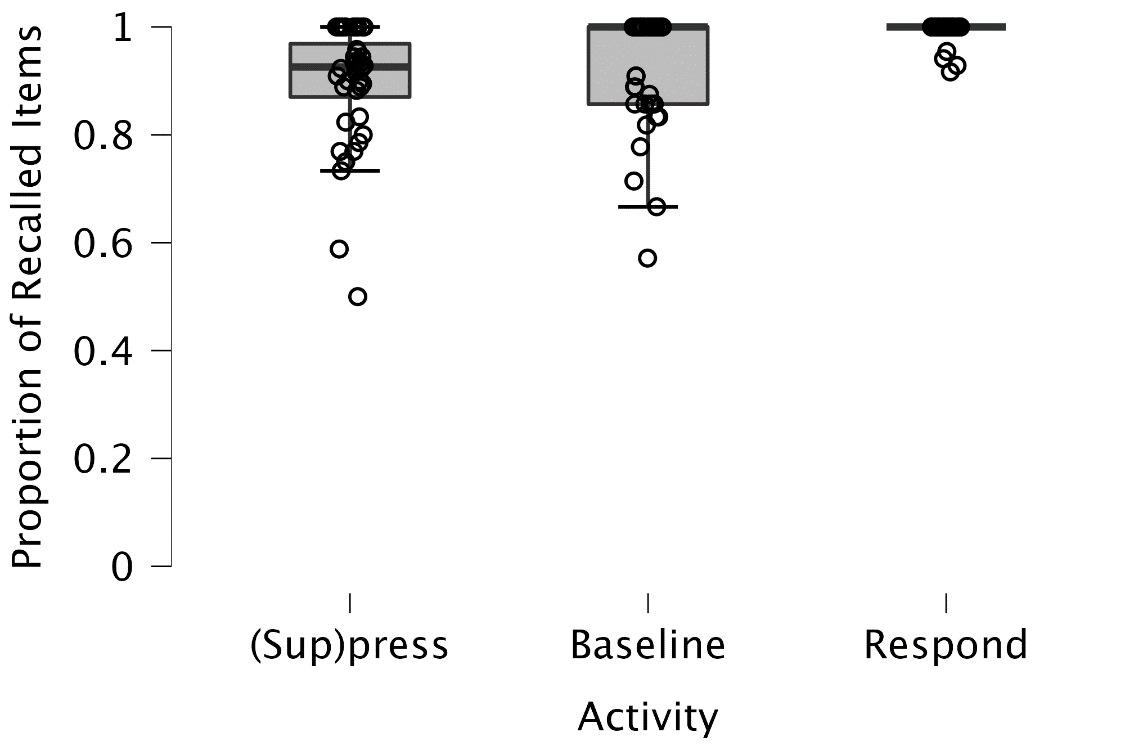
*

*
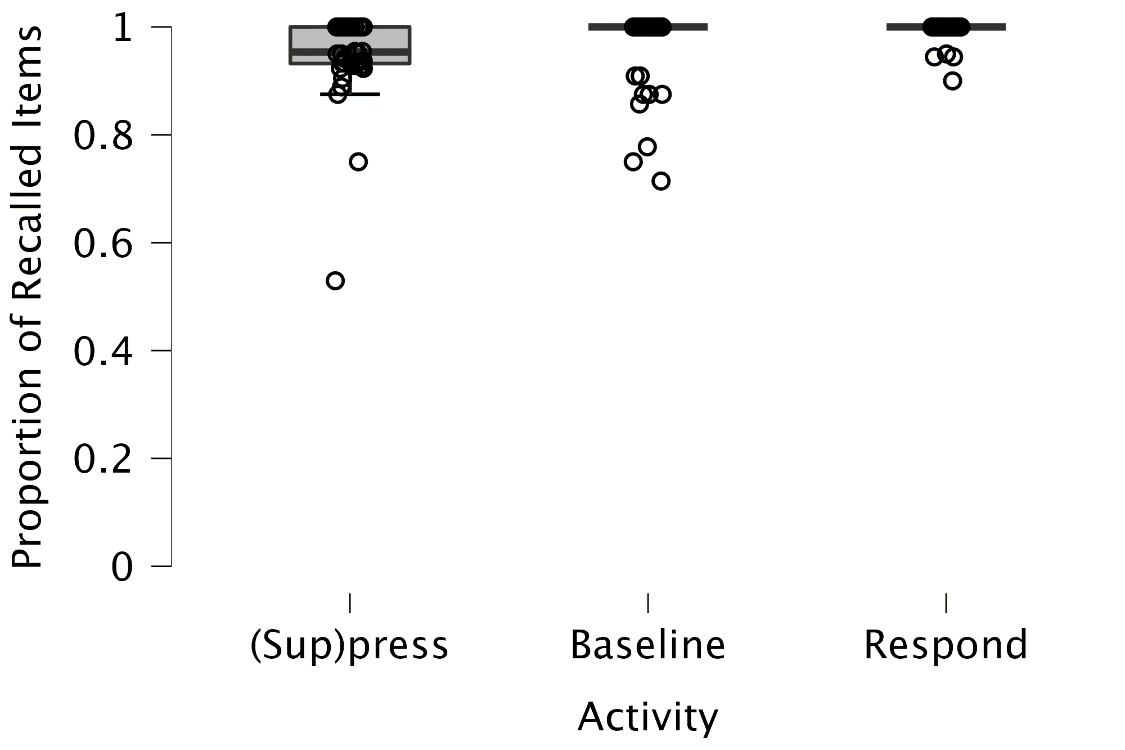
*

Table C2

*Mean (Standard Error) Same Probe Test recall proportion for Experiment 2 for the No-Think group and Press Spacebar group for Baseline, (Sup)press, and Respond*

|  |  | Condition | | |
| --- | --- | --- | --- | --- |
|  |  | (Sup)press | Baseline | Respond |
| Group | No-Think | 0.894 *(0.018)* | 0.924 *(0.017)* | 0.994 *(0.003)* |
|  | Press Spacebar | 0.947 *(0.013)* | 0.964 *(0.012)* | 0.993 *(0.003)* |
